# The Different Facets of Triclocarban: A Review

**DOI:** 10.3390/molecules26092811

**Published:** 2021-05-10

**Authors:** Domenico Iacopetta, Alessia Catalano, Jessica Ceramella, Carmela Saturnino, Lara Salvagno, Ileana Ielo, Dario Drommi, Elisabetta Scali, Maria Rosaria Plutino, Giuseppe Rosace, Maria Stefania Sinicropi

**Affiliations:** 1 Department of Pharmacy, Health and Nutritional Sciences, University of Calabria, 87036 Arcavacata di Rende, Italy; domenico.iacopetta@unical.it (D.I.); jessicaceramella@gmail.com (J.C.); s.sinicropi@unical.it (M.S.S.); 2 Department of Pharmacy-Drug Sciences, University of Bari Aldo Moro, 70126 Bari, Italy; lara.salvagno89@gmail.com; 3 Department of Science, University of Basilicata, 85100 Potenza, Italy; carmela.saturnino@unibas.it; 4 Spinoff TNcKILLERS, Viale dell’Ateneo Lucano 10, 85100 Potenza, Italy; 5 Institute for the Study of Nanostructured Materials, ISMN-CNR, Palermo, Department of ChiBioFarAm, University of Messina, Villa S. Agata, 98166 Messina, Italy; ileana.ielo@ismn.cnr.it (I.I.); mariarosaria.plutino@cnr.it (M.R.P.); 6 Department of ChiBioFarAm, University of Messina, Villa S. Agata, 98166 Messina, Italy; ddrommi@unime.it; 7 Department of Health Sciences, Magna Graecia University, 88100 Catanzaro, Italy; elisabettascali@libero.it; 8 Department of Engineering and Applied Sciences, University of Bergamo, 24044 Dalmine, Italy; giuseppe.rosace@unibg.it

**Keywords:** antimicrobials, diarylureas, bis-arylureas, triclocarban, triclocarban analogues, metabolites, TCC

## Abstract

In the late 1930s and early 1940s, it was discovered that the substitution on aromatic rings of hydrogen atoms with chlorine yielded a novel chemistry of antimicrobials. However, within a few years, many of these compounds and formulations showed adverse effects, including human toxicity, ecotoxicity, and unwanted environmental persistence and bioaccumulation, quickly leading to regulatory bans and phase-outs. Among these, the triclocarban, a polychlorinated aromatic antimicrobial agent, was employed as a major ingredient of toys, clothing, food packaging materials, food industry floors, medical supplies, and especially of personal care products, such as soaps, toothpaste, and shampoo. Triclocarban has been widely used for over 50 years, but only recently some concerns were raised about its endocrine disruptive properties. In September 2016, the U.S. Food and Drug Administration banned its use in over-the-counter hand and body washes because of its toxicity. The withdrawal of triclocarban has prompted the efforts to search for new antimicrobial compounds and several analogues of triclocarban have also been studied. In this review, an examination of different facets of triclocarban and its analogues will be analyzed.

## 1. Introduction

**Figure 1:**
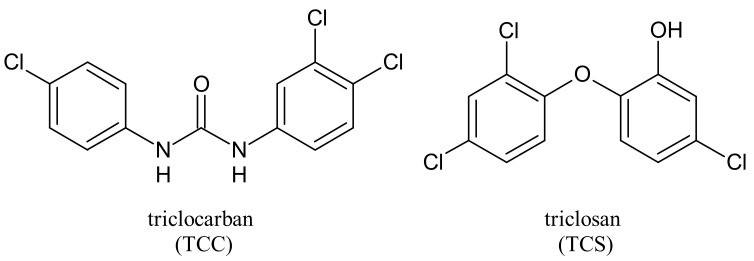
Triclocarban and triclosan.

Triclocarban (TCC, Figure 1) is a highly effective and broad-spectrum antimicrobial and antiseptic agent that has been successfully used in personal care products for over 60 years [1,2]. It is often mentioned along with its congener triclosan (TCS, Figure 1). TCC is a diphenyl urea (*N*-(4-chlorophenyl)-*N*′-(3,4-dichlorophenyl)urea), whereas TCS is an ether (2,4,4′-trichloro-2′-hydroxydiphenyl ether). Although they substantially differ in their structures, TCC and TCS are often taken together probably because of their polychlorinated structure. They even share three chlorine atoms in their structures [3,4,5].

In this review, we will focus our attention on TCC, a small molecule also named 3,4,4′-trichlorocarbanilide that belongs to the class of diarylures or bis-arylureas. This interesting class of compounds has been recently reviewed by our research group as anticancer agents [6], and then, due to their multiple actions [7,8,9,10,11], they were also suggested for repositioning to antimicrobial agents and/or potential treatment for new pandemics, as COVID-19 [12,13]. TCC is a common ingredient in personal care products, especially dermal cleaning products such as antibacterial bar/Liquid soaps, body lotions, deodorants, detergents, medical disinfectants, aftershave soaps, hand sanitizers, toothpaste, handwash and mouthwash, body washes, cleansing lotions, baby teethers and wipes for its sanitizing properties and detergents [14,15,16,17]. It acts as a fungicide and preservative in air fresheners, fabric and leather finishing agents [18]. Its concentration in the products can be as high as 1.5% [19,20], even if its use has been approved by the European Union (EU) in a concentration of 0.2% [21].

Since its advent in 1957 [22], TCC has been produced and used on a large scale, and its consumption from personal care products in the United States reached 500,000–1,000,000 pounds per year [23]. The 2013–2014 National Health and Nutrition Examination Survey showed that 36.9% of the urine samples in the United States (U.S.) contained TCC [24]. Halden et al. (2017) [25] stated that its utility in healthcare settings is uncontested, whereas benefits to its antimicrobial activity are few to none. A 2003 report by the U.S. Centers for Disease Control and Prevention Healthcare Infection Control Practices Advisory Committee concluded that “no evidence is available to suggest that use of [antimicrobial-impregnated articles and consumer items bearing antimicrobial labeling] will make consumers and patients healthier or prevent disease” [26].

TCC belongs to pharmaceutical and personal care products (PPCPs) that comprise large and diversified groups of chemicals, including prescription and over-the-counter drugs and cleaning agents [27]. Unfortunately, PPCPs are often discharged into wastewater treatment plants (WWTPs) via excretion with urine and feces as parent compounds conjugated compounds, or metabolites, and through washing or direct disposal. TCC was demonstrated to be an endocrine disruptor that is hardly biodegradable, being a halogenated hydrocarbon [28,29]. Its extensive utilization in personal care products and its partial removal during conventional WWTPs led to its consideration as an environmental contaminant [30], placing it on the list of emerging organic contaminants (EOCs). Many studies have reported the occurrence of TCC and its intermediates in wastewater effluent, surface water, biosolid, sediment and soil [31,32]. TCC is ranked, in fact, in the top 10 Contaminant of Emerging Concern (CEC) occurrence [31,33,34]. Once conveyed to wastewater treatment plants, PPCPs can remain unchanged or undergo a partial or complete transformation during wastewater treatment processes before being discharged into the environment via effluent and biosolids for land application. Land application of biosolids, the end product of wastewater treatment plants, may be a potential important route through which PPCPs enter the environment [35]. PPCPs biodegradation is a potential removal mechanism, as well [36] and in a recent study TCC aerobic biodegradation was demonstrated to occur slowly (i.e., half-life value > 165 days) [37]. Although PPCPs in the effluent and biosolids of water resource recovery facilities (WRRFs) are currently not regulated, public interest has led the Metropolitan Water Reclamation District of Greater Chicago to monitor for 11 PPCPs in the influent, effluent, and biosolids.

Armstrong et al. studied how WRRFs can influence concentrations before biosolids land application [38]. A critical comprehensive review on TCC as a contaminant has been recently reported [39]. Environmental TCC could be efficiently taken up by food crops, leading to the bioaccumulation of TCC and potential human exposure through food consumption. Some common food crops, such as broccoli, potato, beet, cabbage, and pepper, can accumulate >100 ppm TCC in the root tissues, and onions can accumulate >800 ppm TCC in the bulbs [23]. Human exposure to TCC may also be due to daily supplies, drinking water and dust [26]. As a result, its detection is frequent in human tissue, such as fingernails and body fluids, including blood, urine and seminal plasma [40,41,42,43], thus causing a potential hazard to human health. It is noteworthy that TCC may represent a risk for children, as it has been recently found in urine of Brazilian children and its use also seems to be associated to DNA damage [44]. Human exposure to triclocarban is widely studied in several Asian countries, especially in Vietnam, Kuwait, and Japan [45]. In 2016, the U.S. Food and Drug Administration (FDA) issued a final rule establishing that 19 specific ingredients, including TCC and TCS, were no longer generally recognized as safe and effective, and prohibited companies from marketing soaps as antibacterial containing one or more of these ingredients [46]. This rule came into effect since September 2017 [47]. Though banned in consumer wash products in the U.S., triclocarban is still used in other countries [48]. The EU banned TCS from all human hygiene biocidal products starting from January 2017 [49], whereas China has not regulated TCS and TCC in these products yet [50]. Given the importance of these issues, this review will focus on the activity, occurrence, metabolism, toxicity of TCC and several analogues described in the literature, as well as on compounds potentially effective and usable as an alternative to TCC.

## 2. Metabolism and Transformation Products of TCC

**Figure 2:**
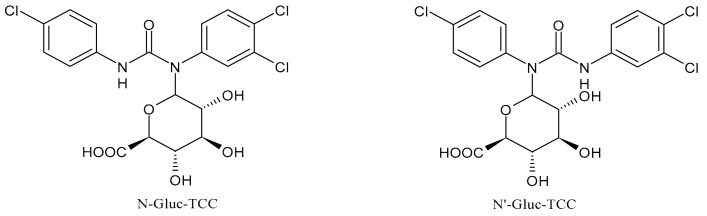
Glucuronide transformation products of triclocarban.

Once applying the TCC-containing products on the skin, they can enter the human body, be metabolized and exert a potential risk for the human health [51]. Indeed, it has been detected in human blood (0.45 ng/mL) and urine (3.85 ng/mL) [52]. Several studies have indicated that a significant portion of TCC in soaps is percutaneously absorbed by humans during and after showering. In humans, it has been estimated that the 0.6% of TCC (approximately 70 μg) can be absorbed, based on the urinary excretion of TCC metabolites [53]. TCC is also present in sanitary pads, panty liners, and tampons, but the transfer rates are not known [54]. TCC exposure may also occur via consumption of water or food [23] and it has been reported that in the maternal and umbilical cord sera it reached the values of 2.75 and 0.82 μg/L, respectively [41]. TCC may undergo phase I and phase II metabolism [55]. The main metabolites detected in human and monkey urine, accounting for 25% of TCC elimination products, result from direct *N*-glucuronidation at one of the nitrogen atoms of the urea moiety of TCC giving N-Gluc-TCC and N’-Gluc-TCC (Figure 2) [56].

**Figure 3:**
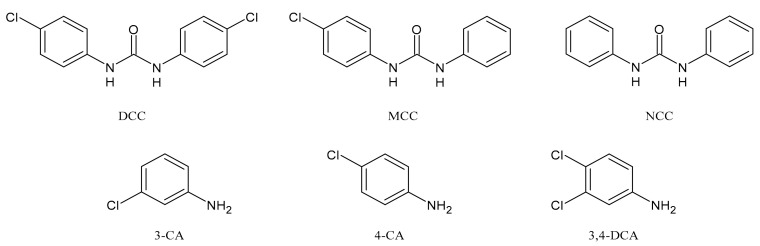
Dechlorinated transformation products of triclocarban.

TCC dechlorination reactions lead to carbanilides (Figure 3) including 4,4′-dichlorocarbanilide (DCC), 1-(3-chlorophenyl)-3-phenylurea (MCC), and carbanilide (NCC), or either biologically or abiotically to 4-chloroaniline (4-CA) [57]. Miller et al. (2010) suggested, for the first time, the occurrence of reductive dechlorination of TCC in estuarine sediments [58]. However, it is unclear whether TCC dechlorination was limited to the sediment environment or whether the WWTPs also contribute to the mitigation of TCC contamination through its dechlorination. Pycke et al. (2014) showed that the anaerobic digestion contribution to partial TCC dechlorination was limited (0.4−2.1%) and described toxic chloroanilines, including 3-chloroaniline (3-CA), and/or 3,4-dichloroaniline (3,4-DCA, Figure 3) [59].

**Figure 4:**
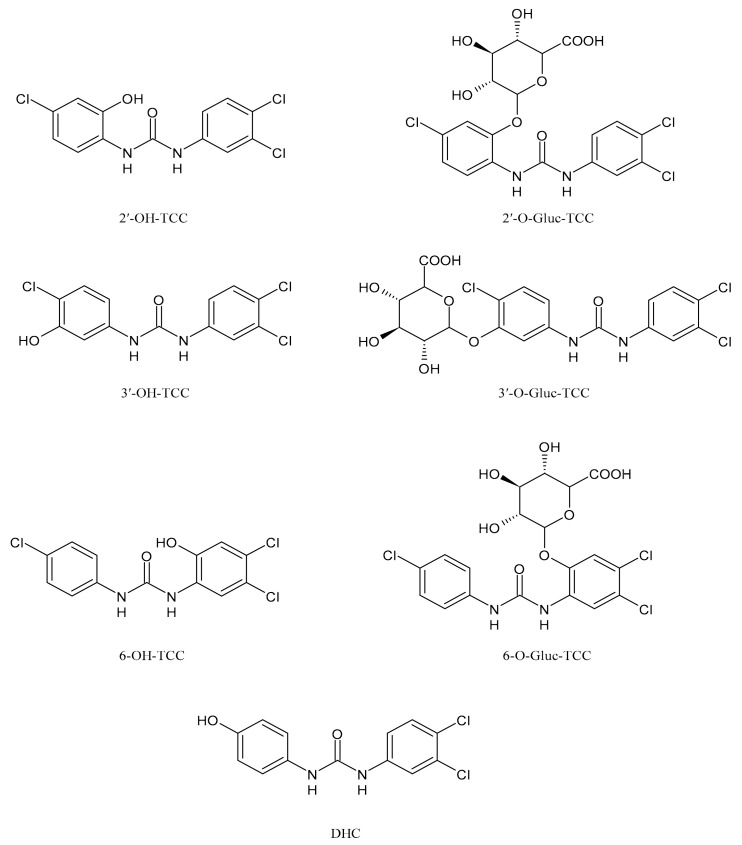
Structures of hydroxylated TCC transformation products and their glucuronides.

TCC is also metabolized by cytochrome P450 enzymes to three hydroxylated species, namely 2′-hydroxytriclocarban (2′-OH-TCC), 3′-hydroxytriclocarban (3′-OH-TCC) and 6-hydroxytriclocarban (6-OH-TCC) (Figure 4) with the *ortho*-hydroxylated species, 2′-OH-TCC and 6-OH-TCC, as main metabolites [53,59]. All metabolites may undergo extensive phase II metabolism, and the glucuronic acid conjugates of the hydroxylated TCC species (2′-*O*-Gluc-TCC, 3′-*O*-Gluc-TCC and 6-*O*-Gluc-TCC) account for the majority of TCC metabolites in mammalian bile and in fishes [60,61]. However, the UDP-glucuronosyltransferases (UGTs) involved in the conjugation of TCC and its metabolites have not been completely defined, as well as the biochemistry and kinetics of the conversion. Schebb et al. 2012 [56] showed that all the major oxidative metabolites of TCC are rapidly conjugated with glucuronic acid by microsomes from the liver, kidney, and intestine. A wide variety of UGTs has high affinity for the hydroxylated TCC metabolites, with high activities, particularly for UGT1A7, UGT1A8, and UGT1A9. Zhang et al. (2020) [62] recently reported another hydroxylated metabolite of TCC called DHC (3′,4′-dichloro-4-hydroxycarbanilide).

## 3. Biological Activity of TCC

Antimicrobial resistance, that is the progressive process by which microbes, such as bacteria, through evolutionary, environmental, and social factors develop the ability to become resistant to drugs that were once effective at treating them, is a threat from which no one can escape [63]. It is a slow but inexorable public health danger that has been defined a “wicked problem”. It has been estimated that globally approximately 700,000 deaths are attributed annually to antimicrobial resistance and this could rise to 10 million deaths per year by 2050 [64]. Intensive efforts are underway worldwide to develop new antimicrobial agents. TCC is an antibacterial and antiseptic agent. The antibacterial activity is generally tested by the determination of the minimum inhibitory concentrations (MICs). According to the FDA guidelines, Kim et al. (2016) [65] stated that MICs are not relevant in this case because consumers are exposed to antiseptic products for a very short time, whereas MIC tests require a long exposure time (at least one day) [66]. Thus, the authors compared the bactericidal effects of plain and antibacterial soaps containing 0.3% TCC. The study was carried out against ten Gram-positive and ten Gram-negative bacterial strains after exposure at 22 °C and 40 °C for 20 s. Gram-negative bacteria were more susceptible to both soaps than Gram-positive bacteria. The authors found no significant difference between the effects of plain and medicated soaps at either temperature, with the only exception of *Enterococcus faecalis* ATCC 19433 at 40 °C. The presence of TCC in soap did not lead to a significant reduction in bacterial levels during the use [65]. *E. faecalis* is a bacterium tragically famed to be part of the top agents responsible for nosocomial infections and is the third most frequent cause of the infective endocarditis (IE), a disease with high morbidity and mortality [67,68].

Another study of the effect of the antibacterial soap Santex, containing TCC, was carried out in a rural Malagasy population that practices subsistence agriculture in the absence of electricity and running water [69]. The authors found that the antibacterial soap influenced the structure of microbial communities, and that these changes persist for at least two weeks, suggesting that antibacterial products may have a lasting impact on skin microbes. Recently, Pujol et al. (2018) [70] determined the MIC value for TCC against *Staphylococcus aureus* ATCC 12600 (MIC = 0.5 µg/mL) and found that it was the same as ciprofloxacin. In a successive paper, the MIC value for TCC was 16 µg/mL against *S. aureus* ATCC 29213 and 6538P (versus 0.5–2 µg/mL of norfloxacin) [71]. Although TCC has been widely used as an antimicrobial for over 50 years, it was only recently that concerns were raised about its endocrine disruptive properties. In 2008, Chen et al. [72] suggested TCC as an endocrine disruptor that enhanced the action of endogenous hormones (androgens and estrogens) rather than directly activating hormone receptors in vitro and in vivo [73]. However, Cao et al. (2020) recently demonstrated that TCC is able to disrupt the estrogen system via the estrogen-related receptor γ (ERRγ) at human exposure levels, via a fluorescence competitive binding assay. TCC demonstrated higher binding potency with ERRγ than the synthetic ERRγ agonist GSK4716, with a dissociation constant of 96 ± 10 nM [74]. TCC has also been shown to disrupt the gut microbiome in animals and humans, leading to a myriad of effects on health [75].

A systematic review about estrogenic and androgenic activities and offspring growth has been reported some years ago [76]. Recently, it has been suggested that the exposure to a relevant dose of TCC may interfere with the human reproduction. For instance, it was associated with a decrease in gestational age [77] and could have implications for human health [52]. TCC could potently inhibit the human soluble epoxide hydrolase (sEH), involved in the biological regulation of pain, inflammation, and blood pressure [53]. TCC has been also demonstrated to induce oxidative stress and cause biological dysfunctions in both animals and humans [78,79]. A study on mouse oocytes showed that TCC exposure disrupted their maturation affecting the cell cycle progression, cytoskeletal dynamics, oxidative stress, early apoptosis, mitochondria function, and histone modifications in vitro [80]. TCC exposure is also a potential environmental risk factor for colitis and associated colonic diseases [81], indeed the exposure to low-dose of TCC exaggerated the severity of colitis and exacerbated the development of colitis-associated colon tumorigenesis, via gut microbiota-dependent mechanisms [23]. Mechanisms related to the toxicity of TCC have not yet been completely defined. For example, TCC exposure promoted the adipogenesis of preadipocytes and hepatocytes in vitro, resulting in toxic lipid accumulation and down-regulation of antioxidant metabolites in hepatocytes [82]. Moreover, Li et al. (2017) showed that TCC inhibited the human aromatase in vitro [83]. TCC activity seems to be related to the disturbance of fatty acid synthesis and the formation of the cell membrane of microbes [84]. More recently, TCC has been shown to disrupt the gut microbiome in animals and humans [85,86], which, in turn, can have myriad effects on health [87].

## 4. Ecotoxicity of Triclocarban

The extensive use of TCC has led to its massive release into the water body. TCC can accumulate in the roots of plants grown in biosolids-amended soils [88] and earthworms living in treated soils [89], indicating the potential ecological risk. TCC has been detected in biosolids from wastewater treatment [90], and its ability to persist in agricultural soils after the land-application of biosolids, has been demonstrated, with an estimated half-life of 191 days [91]. Recent studies have examined the concentrations of TCC in indoor dusts from different microenvironments in Vietnam and the Southeast Asian region. It was found that the concentration of TCC in the kitchen and bedroom dusts was considerably higher than levels found in the living room samples, probably due to its applications in kitchen utensils, household cleaning reagents, and personal care products [4].

TCC can be partially transformed both biotically and abiotically during the WWTP process but the extent to which this takes place is dependent on the treatment methods utilized by the WWT plants. Additionally, TCC degrades via aerobic biodegradation and photolysis into toxic chlorinated anilines [59]. The fate of TCC and its transformation products in wastewater has been extensively studied by Armstrong et al. [38,57,92]. TCC may interact with nitrifying cultures, which are important microbial communities in wastewater treatment plants; however, this interaction has not been totally clarified. Bian et al. (2020) [93] recently reported that nitrifying cultures may remove TCC mainly by inhomogeneous multilayer adsorption, with hydroxyl, amide and polysaccharide seemed to be the main adsorption sites.

On the other end, TCC significantly deteriorated the settleability and performance of nitrifying cultures and inhibited nitrifiers, especially *Nitrospira* sp. Wang et al. (2021) [94] found that about the 71.2–79.4% of TCC was removed by denitrifying sludge in stable operation when its concentration was between 1 and 20 mg/L. Moreover, short-term exposure did not alter physicochemical properties of denitrifying cultures, while long-term exposure deteriorated the settleability, dewaterability, flocculability, and hydrophobicity of denitrifying biomass. It was observed that 20 mg/L of TCC decreased the denitrification efficiency by 70% in long-term operation TCC is usually found more toxic than TCS to aquatic invertebrates and fishes for both short and long-term exposures [95,96]. The potential risk of TCC to the environment has been recently demonstrated by studies of its toxicological effects on the nematode *Caenorhabditis elegans*, which represents an excellent model organism for toxicological studies. TCC induced significant systemic toxic effects in *C. elegans* from apical endpoints to molecular level responses. Lethal toxicity occurred at mg/L levels and sublethal toxicity occurred at µg/L levels [97].

TCC has been reported to be a thyroid disruptor, as well [98]. Wu et al. (2016) [99] studied the effects of TCC on sodium/iodide symporter (NIS)-mediated iodide uptake and the expression of genes involved in thyroid hormone (TH) synthesis in rat thyroid follicular FRTL-5 cells, and on the activity of thyroid peroxidase (TPO) using rat thyroid microsomes. TCC was found to inhibit NIS-mediated iodide uptake in a concentration-dependent manner, acting as a non-competitive inhibitor of NIS, both in the short- and long-term. Recently, studies on the potential hepatotoxic risks of TCC exposure have been reported [62]. In normal hepatocytes, TCC created a prooxidant hepatic environment as evidenced by the decrease of glutathione metabolism and overproduction of reactive oxygen species (ROS), leading to DNA damage and lipid peroxidation. Moreover, TCC treatment may promote liver tumor progression, which resulted from the enhancement of GSH metabolism, glycolysis, and glutaminolysis in cancerous hepatocytes. Hepatic cytotoxicity was also related to its metabolites (6-OH-TCC > 2′-OH-TCC > 3′-OH-TCC > DHC, with EC_50_ values of 2.42, 3.38, 7.38, and 24.8 μM, respectively, in 48 h-treated normal cells).

Junior et al. (2020) [100] carried out studies on TCC toxicity assessments in *Eisenia andrei* earthworms through acute, avoidance and chronic tests following cytotoxicity, antioxidant system, i.e., catalase (CAT), glutathione-*S*-transferase (GST), glutathione (GSH), lipid peroxidation (LPO), and DNA damage (comet assay) evaluations. Chronic exposure to TCC led to reduced CAT and GST activities, decreased GSH levels and increased LPO in exposed organisms. DNA damage was observed after 45 days from a 1 mg/kg dose of TCC, suggesting a toxicological potential of TCC, mainly during long-term exposures. A recent study by Yawer et al. (2020) [101] analyzed the specific role of testicular gap junctional intercellular communication (GJIC) between adjacent prepubertal Leydig TM3 cells in endocrine disruption and male reproductive toxicity. TCC was a potent GJIC-inhibitors causing 50% inhibition at concentrations lower than 60 μM. TCC significantly increased the metabolic/dehydrogenase activity in the Alamar Blue assay, without significant effects on membrane integrity/esterase activity via carboxyfluorescein diacetate (CFDA) or neutral red uptake (NRU) assays. It decreased the cell viability in alamarBlue and NRU assays without the disruption of the cell membrane in CFDA assay. After a prolonged exposure (24 h), TCC caused a decrease in tight junction protein 1 (Tjp1) and Cx45 protein expression. The disturbance of Leydig cell development and functions during a prepubertal period might contribute to the impaired male reproduction. The *Ochrobactrum* sp. MC22 was isolated and identified as plant growth-promoting bacterium with broad and versatile capability of TCC degradation under aerobic and anaerobic conditions. It is able to detoxify TCC and reduce the adverse effect to legume plants [102]. Recently, the removal of PPCPs from wastewater, including TCC, has been obtained by phytoremediation that is the use of algae to treat wastes or wastewaters [103,104,105]. The co-exposure to ibuprofen and triclocarban in the environment was studied and cytotoxicity data suggested that cell growth processes were significantly affected by the co-exposure, consistently with the apoptosis results. The co-occurrence of the two compounds was demonstrated to have synergistic adverse effects for the environment and human health [106]. Recently, Karthikraj et al. (2020) reported the occurrence of TCC in urine of pets (dogs and cats). TCC was not detected (<0.1 ng/mL) in dog urine, whereas it was found in 6% of cat urine [107].

## 5. Analogues of TCC

Several small molecules analogues of TCC, endowed with different biological activities, have been described in the literature and some of them were recently reviewed in detail by our group [13]. Several analogues of TCC showed antimicrobial activity (Table 1) and among them, flucofuron, an insecticide [108], was tested against *S. aureus* (NCTC 8325 and ATCC 12598) and *Staphylococcus epidermidis* (ATCC 12228; 35984) exhibiting a MIC value of 0.25 mg/L (versus 1 mg/L of vancomycin) [109], whereas the compound PK150 was active against *S. aureus* (NCTC 8325), (MIC = 0.3 μM) [110]. In the work by Pujol et al. (2018) above described [70], one or more chlorine atoms of TCC were reduced and/or replaced by pentafluorosulfanyl groups, bioisosteres of the trifluoromethyl groups. The compound **1** exhibited high potency, broad spectrum of antimicrobial activity against Gram-positive bacteria and high selectivity index, while displaying a lower spontaneous mutation frequency than TCC. The compound **1** was able to remove the biofilm formed in a catheter model of infection by *S. aureus* at the same percentage of TCC, similarly to ciprofloxacin. Hassan et al. (2014) reported a study on diarylureas with activity against bacteria and fungi [111]. Particularly, the compound **2** possessed the antimicrobial activity against the tested Gram-positive *Bacillus subtilis* (NCTC-10400) and Gram-negative *Pseudomonas aeruginosa* (ATCC 10145) and *Escherichia coli* (ATCC 23282), showing mean values of inhibition zones (in mm) of 14.0 *versus* 34.0, 32.0 and 30.0 of erythromycin, respectively. The compounds **3** and **4** described by Sarveswari et al. (2018) [112] showed the same activity of the standard (ciprofloxacin) against the Gram-negative *Proteus mirabilis* (ATCC 19181) (zone of inhibition of 23 and 24 mm, respectively, at a concentration of 200 µg/mL compared to 30 mm of ciprofloxacin). Catalano et al. (2021) [71] described the properties of a series of diarylureas analogues of TCC: six compounds (**5**–**10**) showed the same activity of TCC against *S. aureus* (MIC = 16 µg/mL). Out of the six, particularly interesting were the compounds **5** and **6** that also demonstrated even higher activity than TCC against *E. faecalis* (MIC = 32 µg/mL versus MIC = 64 µg/mL of TCC). Moreover, unlike TCC, they both showed no cytotoxicity towards two human cancer cell models, the human mammary epithelial cells MCF-10A and embryonic kidney epithelial cells Hek-293.

Several other analogues of TCC showed antiparasitic activity (Table 2). Particularly, compounds **11–15** were active, in vitro, against *Schistosoma mansoni* on newly transformed schistosomula (NTS) and adult *S. mansoni* worms [113,114], while the compound **16** was active against juvenile and adult *Schistosoma japonicum* [115]. The compound MMV665852 showed activity against both *S. mansoni*, *S. japonicum*, and *Plasmodium falciparum* (EC_50_ = 1160 nM) [115,116]. Khan et al. (2009) reported a series of analogues of TCC and tested their antiglycation activity in vitro according to a literature protocol. All the compounds were more active than the reference rutin, particularly, the compounds **17** and **18** were the most interesting of the series, showing IC_50_ values of 4.26 µM (versus rutin: 41.9 µM) [117]. The diarylurea CP-214339 was an anti-inflammatory agent acting as inhibitor of a ∆5-desaturase in rodents, thus decreasing the arachidonic acid synthesis [118]. The diarylurea **19** behaved as an antiulcer, exhibiting in vitro activity H^+^/K^+^ ATPase (proton pump) (IC_50_ = 18.4 µg/mL), whereas the compound **20** displayed anti-inflammatory activity (IC_50_ = 20.3 µg/mL) [119]. The compound **21** was studied for its implications in the neuroinflammation, showing activity against the transient receptor potential vanilloid 1 (TRPV1) (*K*_i_ for capsaicin antagonism = 0.56 µM) [120]. Some other products of molecular complication of TCC with various activities have been described in our previous paper [13].

## 6. Conclusions and Perspectives

TCC is a lipophilic antimicrobial compound commonly added to a wide range of household and personal care products due to its well-known sanitizing properties. The addition of antibacterial products to hand soaps is a practice that does not enhance the soap effectiveness and can be counterproductive because could contribute to the antibiotic resistance. Moreover, this agent is incompletely removed by wastewater treatment, thus representing an environmental contaminant. A few years ago, TCC was shown to cause adverse effects on human health and unwanted environmental persistence and bioaccumulation, quickly leading to regulatory bans and phase-outs. In 2016, the FDA banned its use in over-the-counter hand and body washes, but this compound is still approved for use in many other personal care products. As a typical organic pollutant, TCC has become one of the top ten common water pollutants due to its extensive releases through wastewater treatment. Dermal exposure from personal care products is believed to be the main route of human exposure to TCC, whereas the minor routes may include contaminated foods. Considering the numerous studies about toxicity of TCC in adults and children, finding new compounds with antibacterial activity similar, better, or higher than TCC, would be desirable. Several analogues of TCC described in this paper are not endowed with cytotoxicity and, thus, they may represent key starting point to develop new molecules acting as antibacterials, and usable in personal care products for adults and children without encountering the known dangerous toxic effects related to the use of TCC. Considered all these issues and the TCC marketing ban, recently many researchers have been pushed to design and synthesize different analogues that maintain the TCC beneficial properties avoiding the undesired ones.

## Figures and Tables

**Table 1:** Analogues of triclocarban with antibacterial activity.

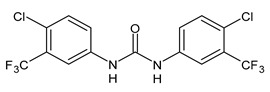	Flucofuron	MIC = 0.25 mg/L (*S. aureus; S. epidermidis*)	Chang et al., 2016 [98]
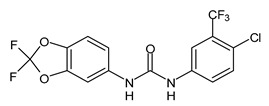	PK150	MIC = 0.3 µM (*S. aureus*)	Le et al., 2020 [110]
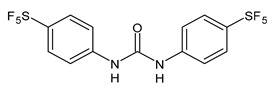	** 1 **	MIC_50_ = 0.05 µg/mL (*S. aureus*)	Pujol et al., 2018 [70]
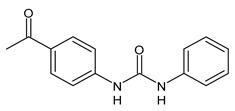	** 2 **	Inhibition zones = 14.0 mm *(B. subtilis* *P. aeruginosa. E. coli)*	Hassan et al., 2014 [111]
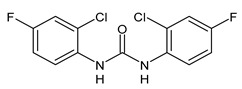	** 3 **	Inhibition zone = 23 mm (*P. mirabilis*)	Sarveswari et al., 2018 [112]
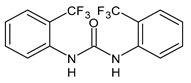	** 4 **	Inhibition zone = 24 mm (*P. mirabilis*)	Sarveswari et al., 2018 [112]
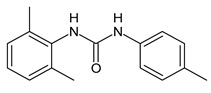	** 5 **	MIC = 16 µM (*S. aureus)* MIC = 32 µM (*E. faecalis*)	Catalano et al., 2021 [71]
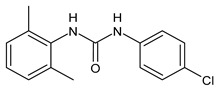	** 6 **	MIC = 16 µM (*S. aureus)* MIC = 32 µM (*E. faecalis*)	Catalano et al., 2021 [71]
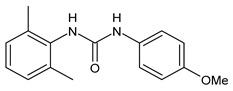	** 7 **	MIC = 16 µM (*S. aureus)*	Catalano et al., 2021 [71]
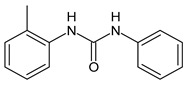	** 8 **	MIC = 16 µM (*S. aureus)*	Catalano et al., 2021 [71]
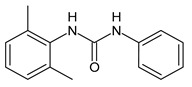	** 9 **	MIC = 16 µM (*S. aureus)*	Catalano et al., 2021 [71]
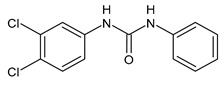	** 10 **	MIC = 16 µM (*S. aureus)*	Catalano et al., 2021 [71]

**Table 2:** Analogues of triclocarban with various activities.

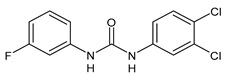	**11**	IC_50_ = 1.3 µM (*S. mansoni* NTS) IC_50_ = 0.7 µM (adult *S. mansoni*)	Cowan et al., 2015 [113]
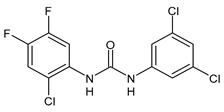	**12**	IC_50_ = 0.2 µM (adult *S. mansoni*)	Cowan et al., 2015 [113]
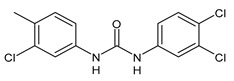	**13**	IC_50_ = 3.6 µM (adult *S. mansoni*)	Cowan et al., 2015 [113]
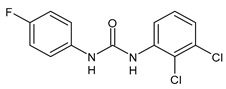	**14**	IC_50_ = 7.0 µM (adult *S. mansoni*)	Cowan et al., 2015 [113]
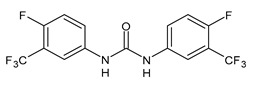	**15**	IC_50_ = 0.15 µM (*S. mansoni* NTS) IC_50_ = 0.19 µM (adult *S. mansoni*)	Wu et al., 2018 [114]
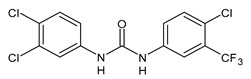	**16**	IC_50_ = 2.5 µM (juvenile *S. japonicum*) IC_50_ = 1.5 µM (adult *S. japonicum*)	Yao et al., 2016 [115]
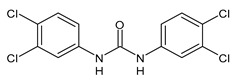	** MMV665852 **	IC_50_ = 4.7 µM (*S. mansoni* NTS) IC_50_ = 0.8 µM (adult *S. mansoni*) IC_50_ = 4.4 µM (juvenile *S. japonicum*) IC_50_ = 2.2 µM (adult *S. japonicum*) EC_50_ = 1160 nM (*P. falciparum*)	Ingram-Sieber et al., 2014 [116] Yao et al., 2016 [115]
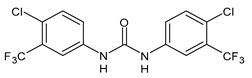	**Flucofuron**	IC_50_ = 2.8 µM (juvenile *S. japonicum*) IC_50_ = 1.5 µM (adult *S. japonicum*)	Yao et al., 2016 [115]
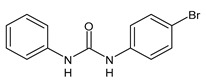	**17**	C_50_ = 4.26 µM (antiglycating activity)	Khan et al., 2009 [117]
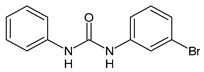	**18**	IC_50_ = 4.26 µM (antiglycating activity)	Khan et al., 2009 [117]
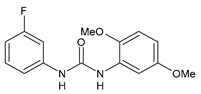	**CP-214339**	IC_50_ = 0.13 µM (Δ5-desaturase inhibitor in rodents)	Obukowicz et al., 1998 [118]
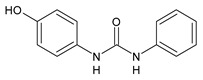	**19**	IC_50_ = 18.4 µg/mL (proton pump inhibition)	Rakesh et al., 2017 [119]
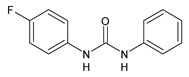	**20**	IC_50_ = 20.3 µg/mL (anti-inflammatory activity in human blood)	Rakesh et al., 2017 [119]
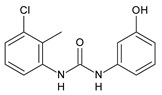	**21**	*K*_i_ = 0.56 µM (TRPV1 capsaicin antagonism)	Feng et al., 2016 [120]

## Abbreviations

*B. subtilis*	*Bacillus subtilis*
CAT	Acatalase
3-CA	3-Chloroaniline
4-CA	4-Chloroaniline
CFDA	Carboxyfluorescein diacetate
CEC	Contaminant of Emerging Concern
*C. elegans*	*Caenorhabditis elegans*
3,4-DCA	3,4-Dichloroaniline
DCC	4,4′-Dichlorocarbanilide
DHC	3′,4′-Dichloro-4-hydroxycarbanilide
*E. coli*	*Escherichia coli*
*E. faecalis*	*Enterococcus faecalis*
EOCs	Emerging Organic Contaminants
ERRγ	Estrogen-related receptor γ
EU	European Union
FDA	Food and Drug Administration
GJIC	Gap junctional intercellular communication
GSH	Glutathione
GST	GLUTATHIONE-S-TRANSFERASE
IE	INFECTIVE ENDOCARDITIS
LPO	LIPID PEROXIDATION
MCC	1-(3-Chlorophenyl)-3-phenylurea
MICs	Minimum inhibitory concentrations
NCC	Carbanilide
NIS	Sodium/iodide symporter
NRU	Neutral red uptake
2′-OH-TCC	2′-Hydroxytriclocarban
3′-OH-TCC	3′-Hydroxytriclocarban
*P. aeruginosa*	*Pseudomonas aeruginosa*
*P. falciparum*	*Plasmodium falciparum*
*P. mirabilis*	*Proteus mirabilis*
PPCPs	Personal care product compounds
*S. aureus*	*Staphylococcus aureus*
*S. epidermidis*	*Staphylococcus epidermidis*
*S. japonicum*	*Schistosoma japonicum*
*S. mansoni*	*Schistosoma mansoni*
sEH	Soluble epoxide hydrolase
TCC	Triclocarban
TCS	Triclosan
TH	Thyroid hormone
Tjp1	Tight junction protein 1
TPO	Thyroid peroxidase
TRVP1	Transient receptor potential vanilloid 1
UGTs	UDP-glucuronosyltransferases
U.S.	United States
WRRF	Water resource recovery facility
WWT	Wastewater treatment
WWTPs	Wastewater treatment plants
